# Supplementary material for: In vitro γ-aminobutyric acid A (GABAA) receptor activity and binding interactions at the α+/γ2– interface of 53 prescription and designer benzodiazepines
Source: Commun Chem. 2026 Apr 7;9:155. doi: 10.1038/s42004-026-02001-x (PMC13068902; doi:10.1038/s42004-026-02001-x)
Supplement: Supplementary file 2 — Supplementary information [file 42004_2026_2001_MOESM2_ESM.pdf]

***In vitro* γ-aminobutyric acid A ($GABA_A$) receptor activity and binding interactions at the α^+/γ_2^- interface of 53 prescription and designer benzodiazepines**

Caitlyn Norman[1,*], Sara I. Liin[2], Amaia Jauregi-Miguel[2,3], Nina E. Ottosson[2,3], Henrik Gréen[1,4]

[1]Division of Clinical Chemistry and Pharmacology, Department of Biomedical and Clinical Sciences, Faculty of Medicine, Linköping University, Linköping, Sweden
[2]Division of Neurobiology, Department of Biomedical and Clinical Sciences, Linköping University, Linköping, Sweden
[3]Chemical Biology Consortium Sweden, Science for Life Laboratory, Linköping University, Linköping, Sweden
[4]Department of Forensic Genetics and Forensic Toxicology, National Board of Forensic Medicine, Linköping, Sweden
*Corresponding author: caitlyn.norman@liu.se

Supplementary Information

SECTION 1: $GABA_A$ assay development
SECTION 2: Comparison of BZD analogs with different base structures
SECTION 3: Comparison of BZD analogs with different substitutions on the fused benzene ring (R_4 in Figure 1)
SECTION 4: Comparison of BZD analogs with different substitutions on the fused thieno ring (R_5 in Figure 1)
SECTION 5: Comparison of BZD analogs with different substitutions at the 2 position on the benzene ring (R_6 in Figure 1)
SECTION 6: Comparison of BZD parent compounds and metabolites and prodrugs and their primary metabolite
SECTION 7: Complete data from the statistical analysis of structure-activity relationships
SECTION 8: Suggested doses for D/BZDs from TripSit

SECTION 1
Assay Development

S1.1. GABA concentration dependence

At the beginning of the assay development, the GABA concentration dependence was examined by testing increasing concentrations of GABA. As shown in Supplementary Figure 1(A) and the resulting dose-response curve in Supplementary Figure 1(B), as expected, increasing concentrations of GABA led to increasing electrical currents.

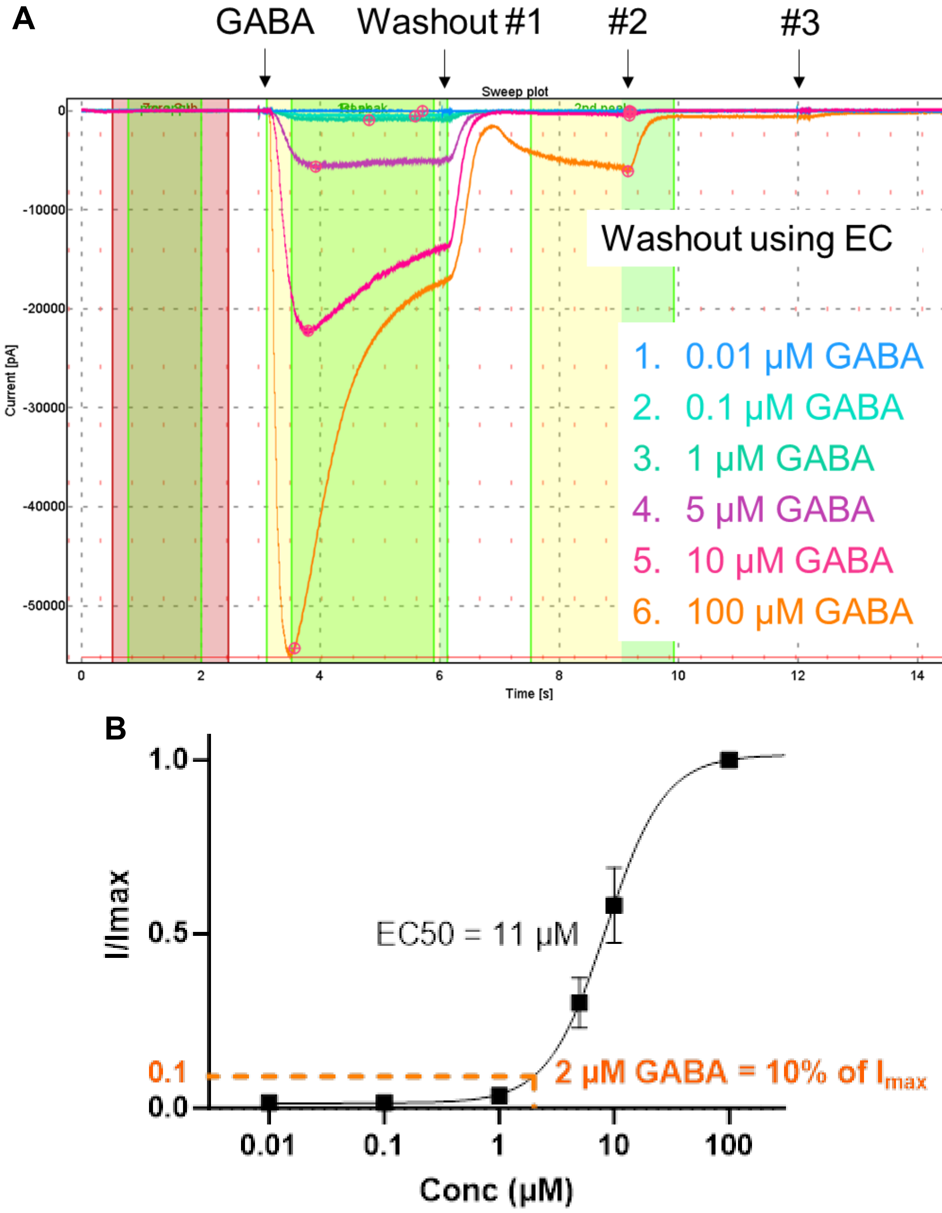

Supplementary Figure 1. (A) Electric currents from $GABA_A$ assay of increasing concentrations of GABA with three washouts using extracellular (EC) solution. (B) Resulting dose-response curve of GABA where 100 µM of GABA is set as the I_{max}. Error bars indicate standard error to the mean (SEM).

Since BZDs are PAMs, meaning they need the presence of GABA in order to activate the $GABA_A$ receptor, a concentration of 2 µM GABA that generates approximately 10% of the maximum current (I_{max}) was chosen as the concentration to use with BZDs. This ensured a stable baseline of GABA. In addition, as there is only a certain amount of electric current that can be recorded from the $GABA_A$ ion channel, using a GABA concentration with a higher electric current would limit the effect of the BZDs that could be recorded and thereby reduce the efficacy of the BZDs.

S1.2. Pre-incubation of the cells

During development of the assay, it was found that activation of the $GABA_A$ receptor by diazepam produced electric currents with negative slopes, as shown in Supplementary Figure 2(A). This indicated that it takes time for the BZDs to bind to the $GABA_A$ receptor and thereby enhance the GABA-induced chloride ion flux, so the full effect of the BZD was not found. To try to address this, the cells were pre-incubated with BZDs for 5 mins before being applied with GABA during the experimental recordings. The time in the presence of GABA was also increased from 3s to 5s. As can be seen in Supplementary Figure 2(B), this resulted in stable electric currents or electric currents with a positive slope, suggesting the full effect of diazepam occurred immediately upon the addition of GABA. The use of pre-incubation also produced a large increase in the efficacy and potency of diazepam, as shown in Supplementary Figure 2(C). Therefore, it was determined that pre-incubation of the cells with the BZDs was necessary to observe the full effect of the drugs.

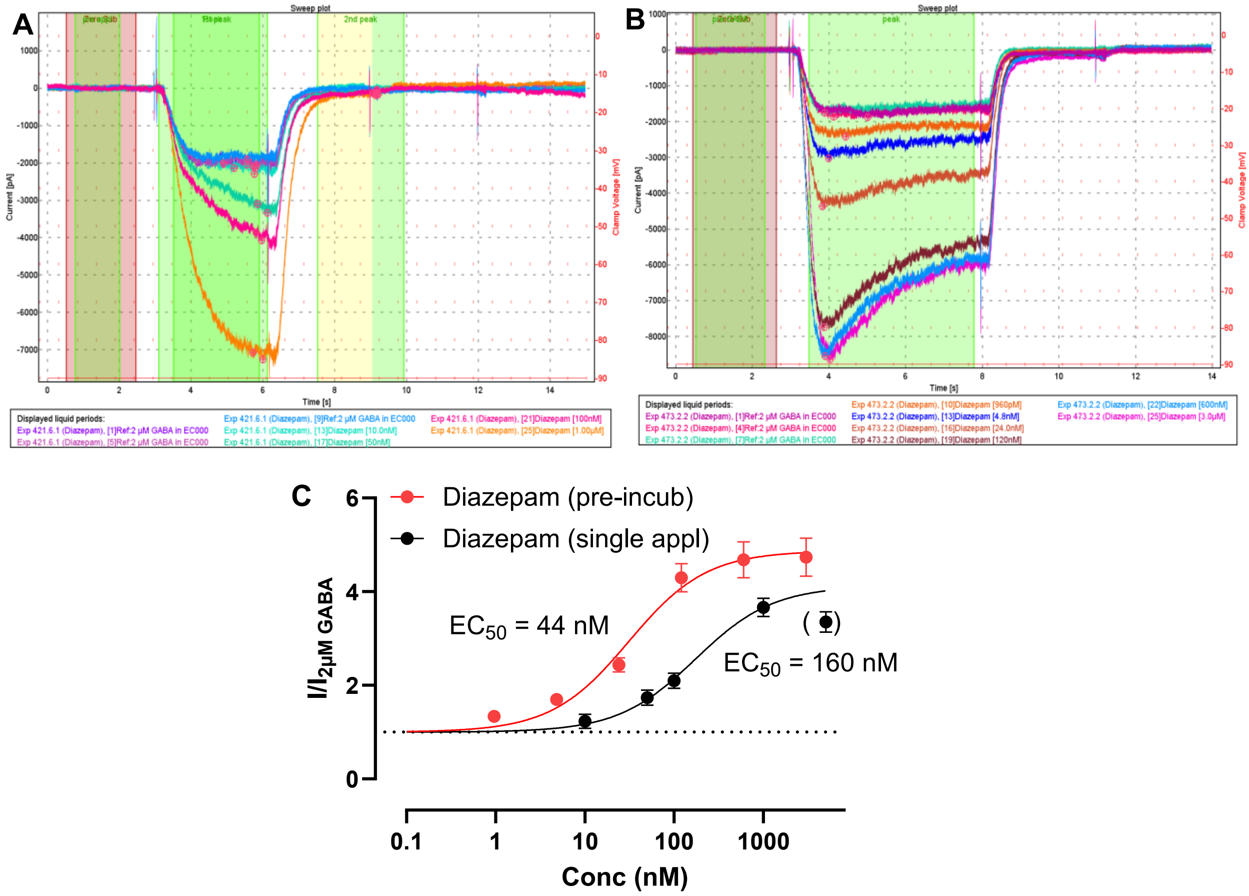

Supplementary Figure 2. (A) Electrical currents from $GABA_A$ assay of diazepam run without pre-incubation. (B) Electrical currents from $GABA_A$ assay of diazepam run with pre-incubation. (C) Concentration-response curves of diazepam run without pre-incubation (single appl) and with pre-incubation (pre-incub). Error bars indicate standard error to the mean (SEM).

S1.3. Change in the osmolarity of the IC solution

The osmolarity of the IC solution was 305-308 mOsm at the beginning of the study; however, the osmolarity was later increased to 310-313 mOsm to improve the stability of the cells in the assay. As shown in Supplementary Figure 3, this did not significantly impact the calculated efficacy or potency values of diazepam, so all data is comparable regardless of the IC solution used.

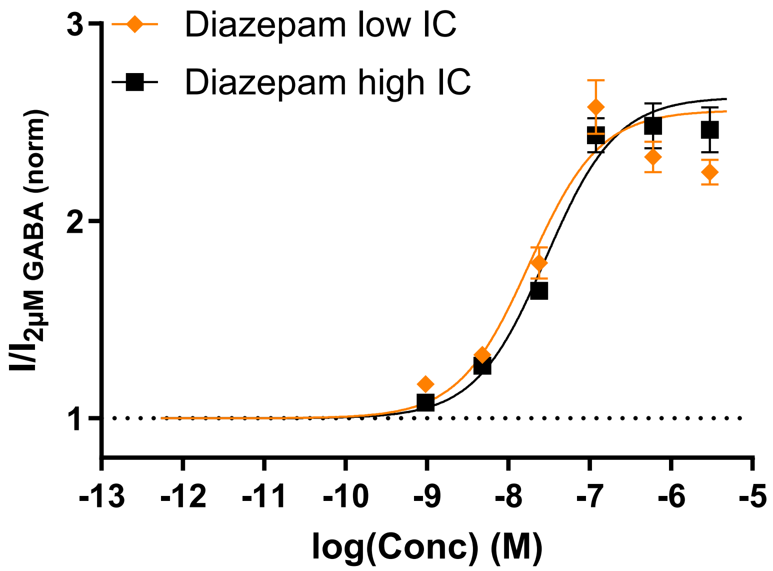

IC (mOsm)	Efficacy (E_{max})			Potency (EC_{50})		
	E_{max}	95% CI	P value	EC_{50}	95% CI	P value
Low (305-308)	2.56	2.35-2.79	0.628	18.1	9.84-31.7	0.221
High (310-313)	2.63	2.47-2.79		29.2	19.4-43.2	

Supplementary Figure 3. Concentration-response curves of diazepam run on the same MTP plate with intracellular (IC) solutions at low osmolarity (305-308 mOsm) or high osmolarity (310-313 mOsm). Error bars indicate standard error to the mean (SEM). A table with the calculated efficacy and potency values with the 95% confidence interval (CI) are also provided. The p values are from unpaired t-tests of the low and high IC diazepam E_{max} and EC_{50} values.

SECTION 2

Comparison of BZD analogs with different base structures

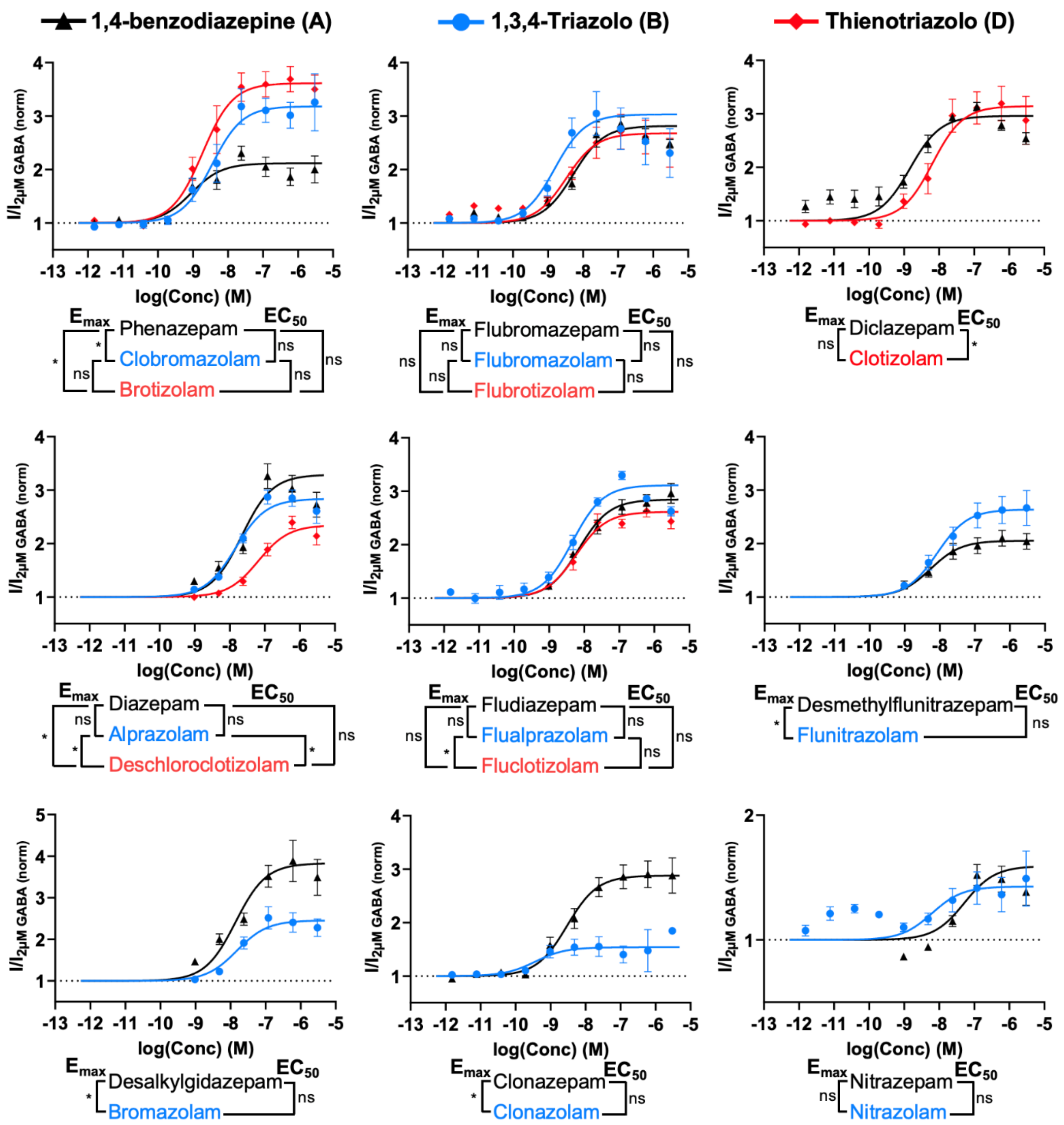

Supplementary Figure 4. Comparison of the *in vitro* $GABA_A$ receptor activity of benzodiazepines that are close analogs with different base structures: 1,4-benzodiazepine (base A in Figure 1), triazolobenzodiazepines (base B in Figure 1), and thienotriazolobenzodiazepines (base D in Figure 1). The data was normalized so the baseline $GABA_A$ (2 μM) activity was at an efficacy of 1, which is shown as a dotted line. Error bars indicate standard error to the mean (SEM). The results of statistical comparisons from Brown-Forsythe and Welch ANOVA tests (α = 0.05) for the efficacy (E_{max}) and potency (EC_{50}) between benzodiazepine analogs with different substitutions are provided where * indicates statistically significant and "ns" indicates not statistically significant. Complete statistical data can be found in Supplementary Table 1.

Comparison of BZD analogs with different base structures, with the structures shown

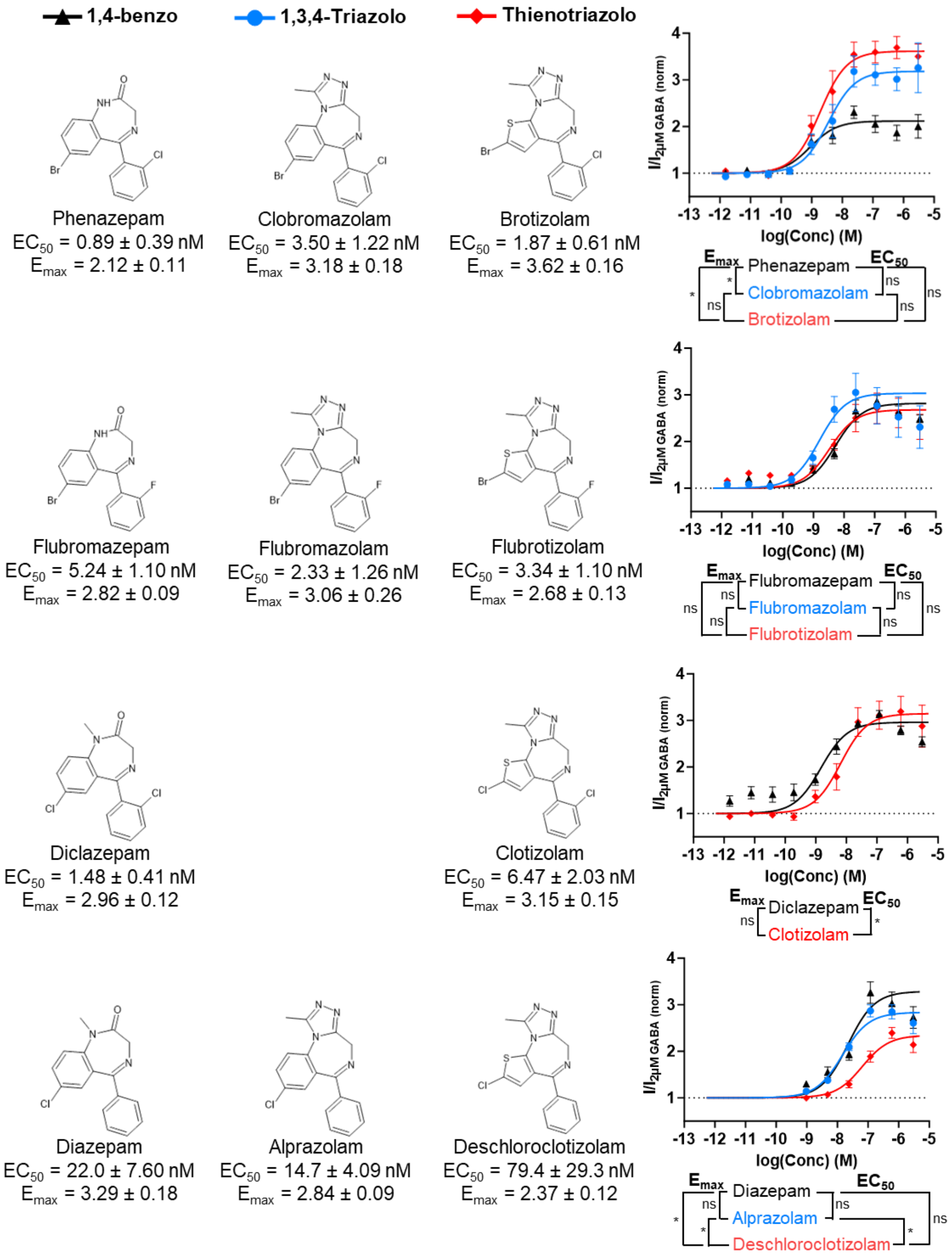

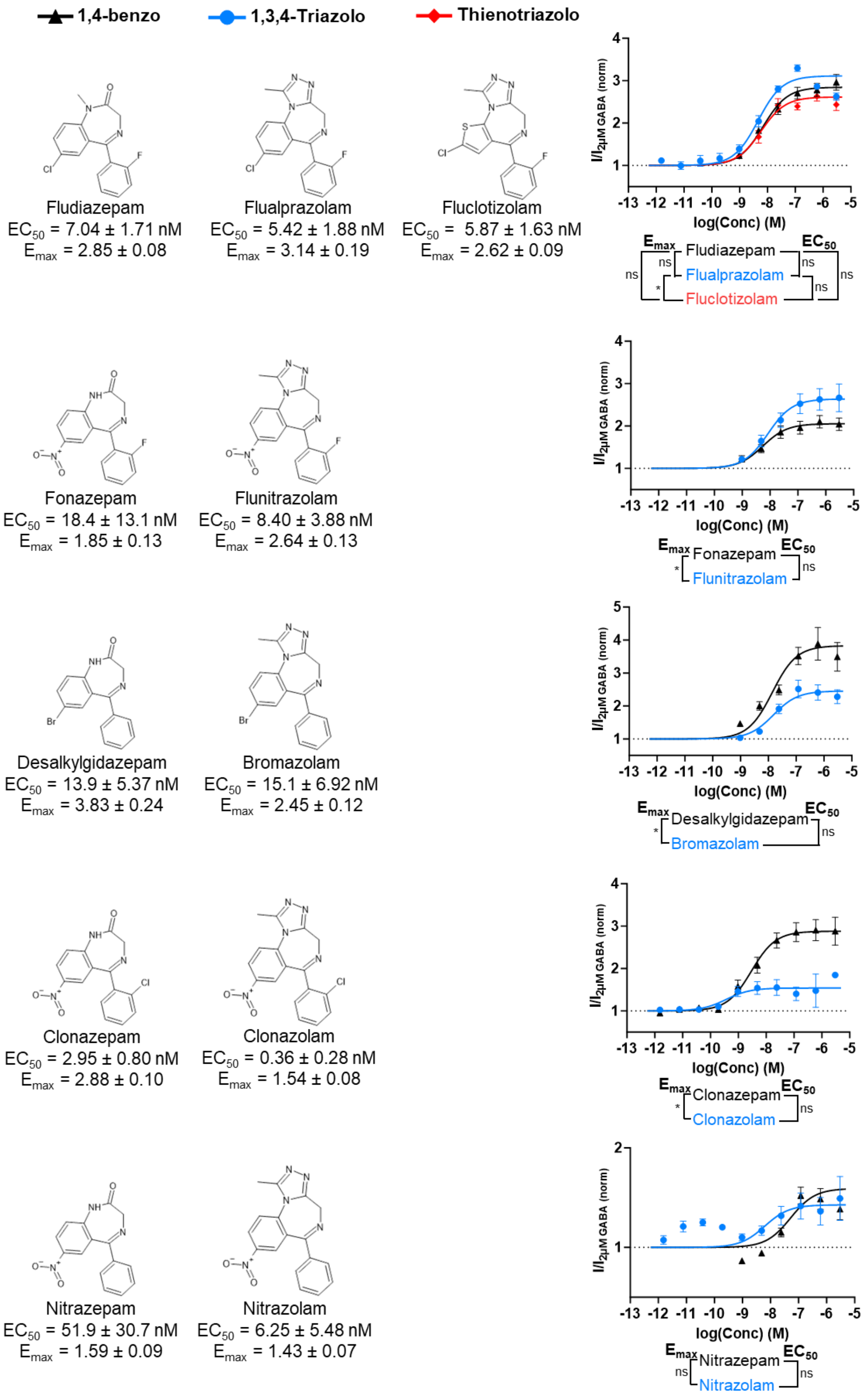

1,4-benzo
1,3,4-Triazolo
Thienotriazolo
Fludiazepam
EC50 = 7.04 ± 1.71 nM
Emax = 2.85 ± 0.08
Flualprazolam
EC50 = 5.42 ± 1.88 nM
Emax = 3.14 ± 0.19
Fluclotizolam
EC50 = 5.87 ± 1.63 nM
Emax = 2.62 ± 0.09
I/I2μM GABA (norm)
log(Conc) (M)
Emax
EC50
Fludiazepam
Flualprazolam
Fluclotizolam
ns
*
Fonazepam
EC50 = 18.4 ± 13.1 nM
Emax = 1.85 ± 0.13
Flunitrazolam
EC50 = 8.40 ± 3.88 nM
Emax = 2.64 ± 0.13
Desalkylgidazepam
EC50 = 13.9 ± 5.37 nM
Emax = 3.83 ± 0.24
Bromazolam
EC50 = 15.1 ± 6.92 nM
Emax = 2.45 ± 0.12
Clonazepam
EC50 = 2.95 ± 0.80 nM
Emax = 2.88 ± 0.10
Clonazolam
EC50 = 0.36 ± 0.28 nM
Emax = 1.54 ± 0.08
Nitrazepam
EC50 = 51.9 ± 30.7 nM
Emax = 1.59 ± 0.09
Nitrazolam
EC50 = 6.25 ± 5.48 nM
Emax = 1.43 ± 0.07

SECTION 3

Comparison of BZD analogs with different substitutions on the fused benzene ring (R_4 in Figure 1)

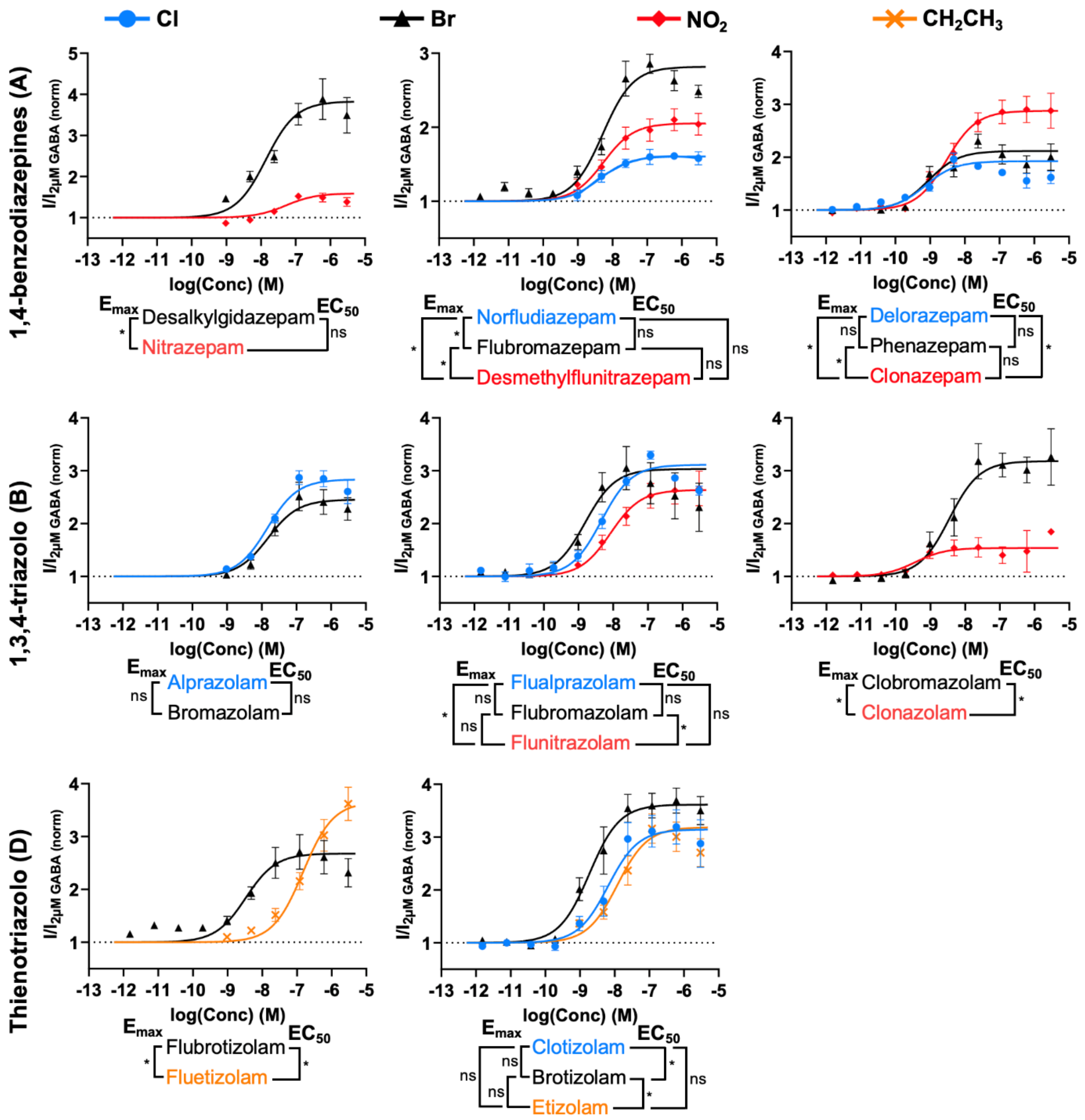

Supplementary Figure 5. Comparison of the *in vitro* $GABA_A$ receptor activity of benzodiazepines that are close analogs with different substitutions at position 7 on the fused benzene ring (R_4) of 1,4-benzodiazepine (base A in Table 1) and triazolobenzodiazepines (base B in Table 1) and substitutions at the 2 position on the fused thieno ring (R_5) of thienotriazolobenzodiazepines (base D in Table 1). The data was normalized so the baseline $GABA_A$ (2 µM) activity was at an efficacy of 1, which is shown as a dotted line. Error bars indicate standard error to the mean (SEM). The results of statistical comparisons from Brown-Forsythe and Welch ANOVA tests ($\alpha = 0.05$) for the efficacy (E_{max}) and potency (EC_{50}) between benzodiazepine analogs with different substitutions are provided where * indicates statistically significant and "ns" indicates not statistically significant. Complete statistical data can be found in Supplementary Table 4.

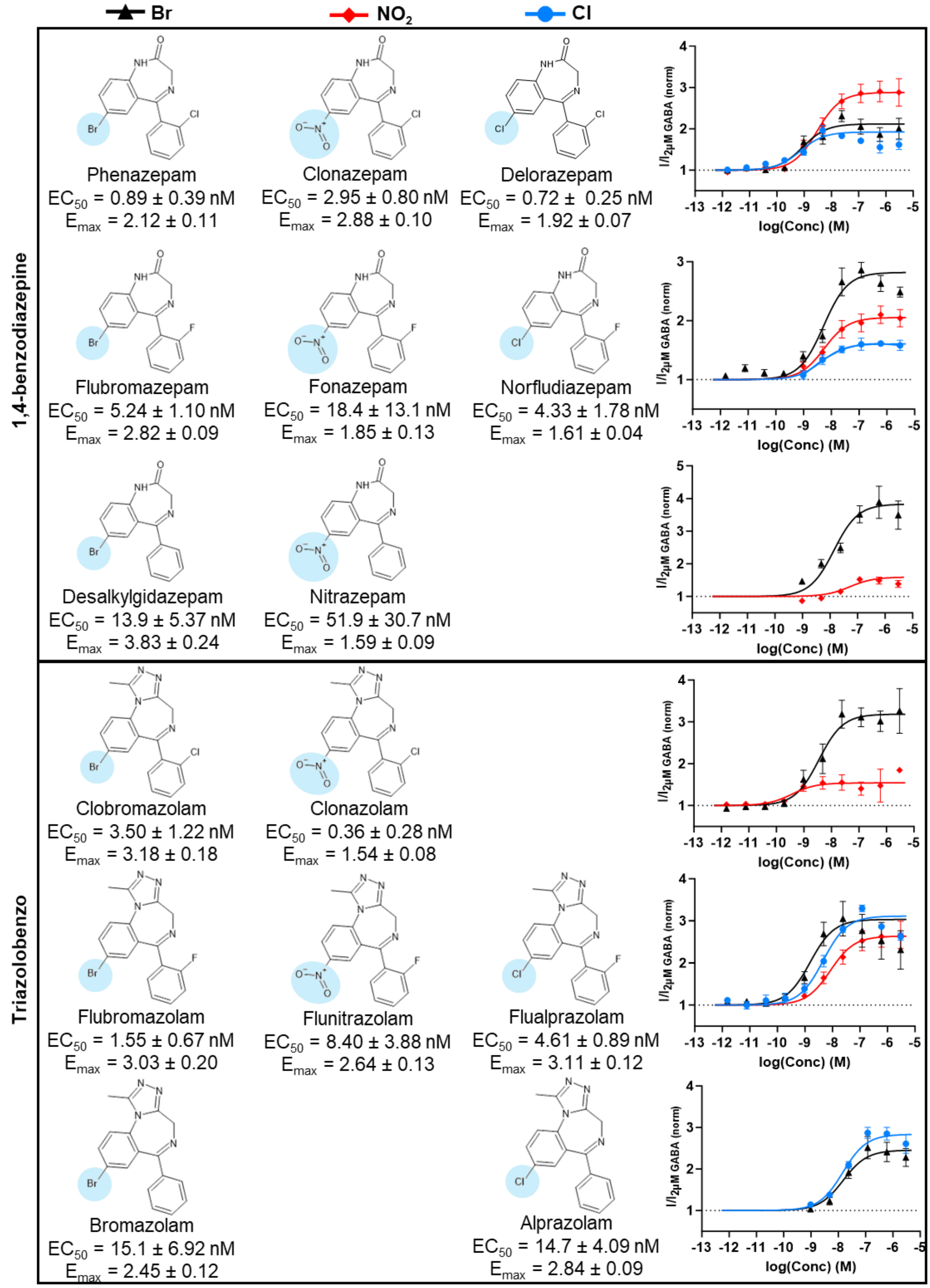

Br
NO_2
Cl
1,4-benzodiazepine
Phenazepam
EC_{50} = 0.89 ± 0.39 nM
E_{max} = 2.12 ± 0.11
Clonazepam
EC_{50} = 2.95 ± 0.80 nM
E_{max} = 2.88 ± 0.10
Delorazepam
EC_{50} = 0.72 ± 0.25 nM
E_{max} = 1.92 ± 0.07
Flubromazepam
EC_{50} = 5.24 ± 1.10 nM
E_{max} = 2.82 ± 0.09
Fonazepam
EC_{50} = 18.4 ± 13.1 nM
E_{max} = 1.85 ± 0.13
Norfludiazepam
EC_{50} = 4.33 ± 1.78 nM
E_{max} = 1.61 ± 0.04
Desalkylgidazepam
EC_{50} = 13.9 ± 5.37 nM
E_{max} = 3.83 ± 0.24
Nitrazepam
EC_{50} = 51.9 ± 30.7 nM
E_{max} = 1.59 ± 0.09
Triazolobenzo
Clobromazolam
EC_{50} = 3.50 ± 1.22 nM
E_{max} = 3.18 ± 0.18
Clonazolam
EC_{50} = 0.36 ± 0.28 nM
E_{max} = 1.54 ± 0.08
Flubromazolam
EC_{50} = 1.55 ± 0.67 nM
E_{max} = 3.03 ± 0.20
Flunitrazolam
EC_{50} = 8.40 ± 3.88 nM
E_{max} = 2.64 ± 0.13
Flualprazolam
EC_{50} = 4.61 ± 0.89 nM
E_{max} = 3.11 ± 0.12
Bromazolam
EC_{50} = 15.1 ± 6.92 nM
E_{max} = 2.45 ± 0.12
Alprazolam
EC_{50} = 14.7 ± 4.09 nM
E_{max} = 2.84 ± 0.09
$I/I_{2\mu M\ GABA}$ (norm)
log(Conc) (M)

SECTION 4

Comparison of BZD analogs with different substitutions on the fused thieno ring (R_5 in Figure 1)

In this section, the structure of each benzodiazepine studied with substitutions on the fused thieno ring (R_5 in Figure 1) is provided with the structural differences highlighted. The concentration-response curves for the compared benzodiazepines are provided.

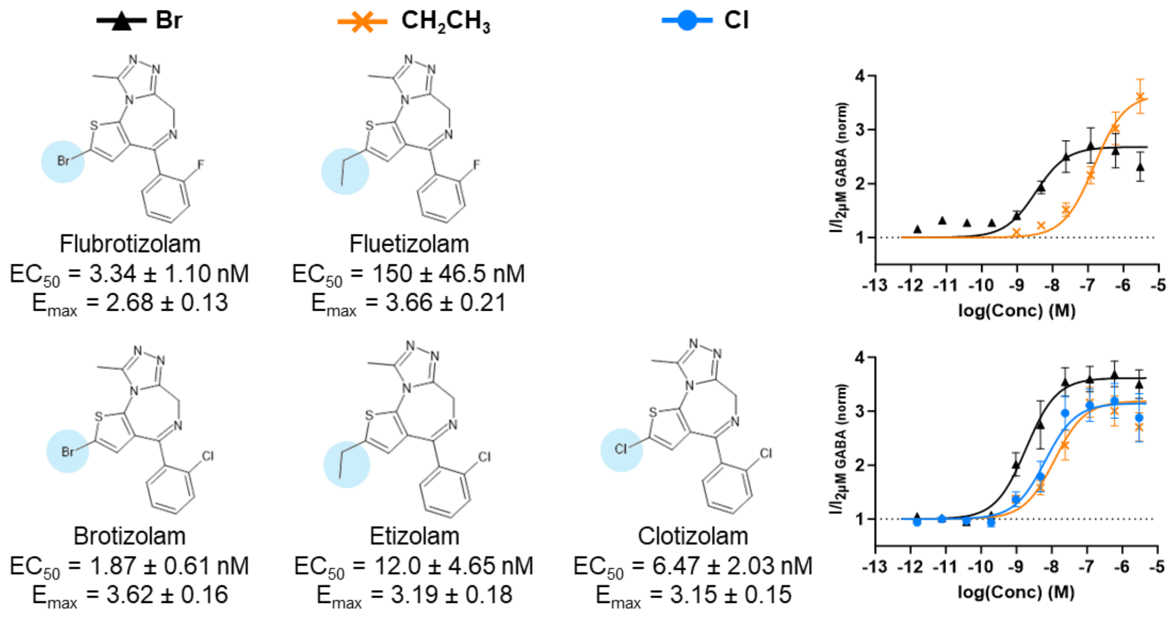

SECTION 5

Comparison of BZD analogs with different substitutions at the 2 position on the benzene ring (R_6 in Figure 1)

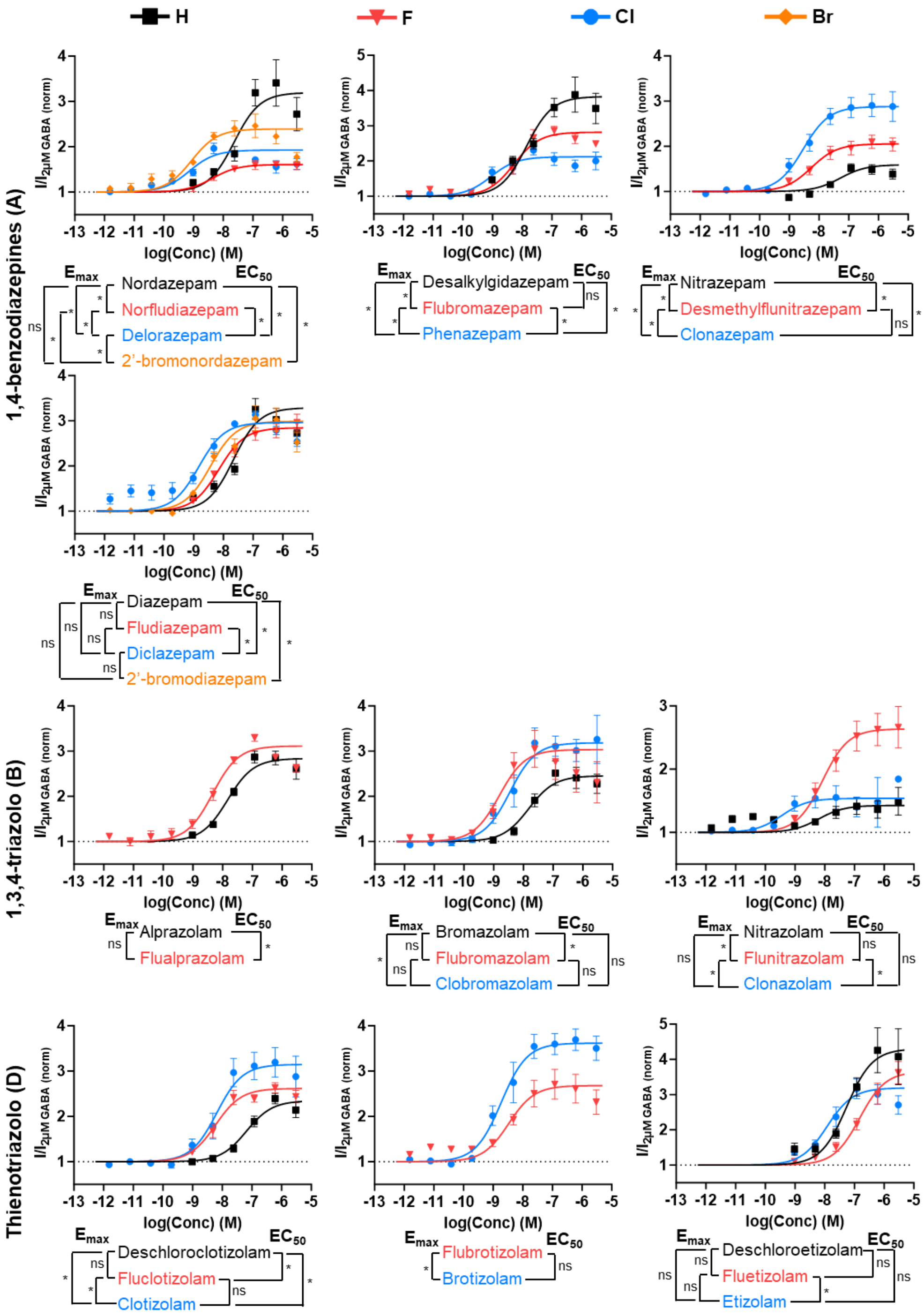

Supplementary Figure 6. Comparison of the *in vitro* $GABA_A$ receptor activity of benzodiazepines that are close analogs with different substitutions at position 2 on the benzene ring (R_6 Table 1). The data was normalized so the baseline $GABA_A$ (2 μM) activity was at an efficacy of 1, which is shown as a dotted line. Error bars indicate standard error to the mean (SEM). The results of statistical comparisons from Brown-Forsythe and Welch ANOVA tests ($\alpha = 0.05$) for the efficacy (E_{max}) and potency (EC_{50}) between benzodiazepine analogs with different substitutions are provided where * indicates statistically significant and "ns" indicates not statistically significant. Complete statistical data can be found in Supplementary Table 3.

Comparison of BZD analogs with different substitutions at the 2 position on the benzene ring (R_6), with the structures shown

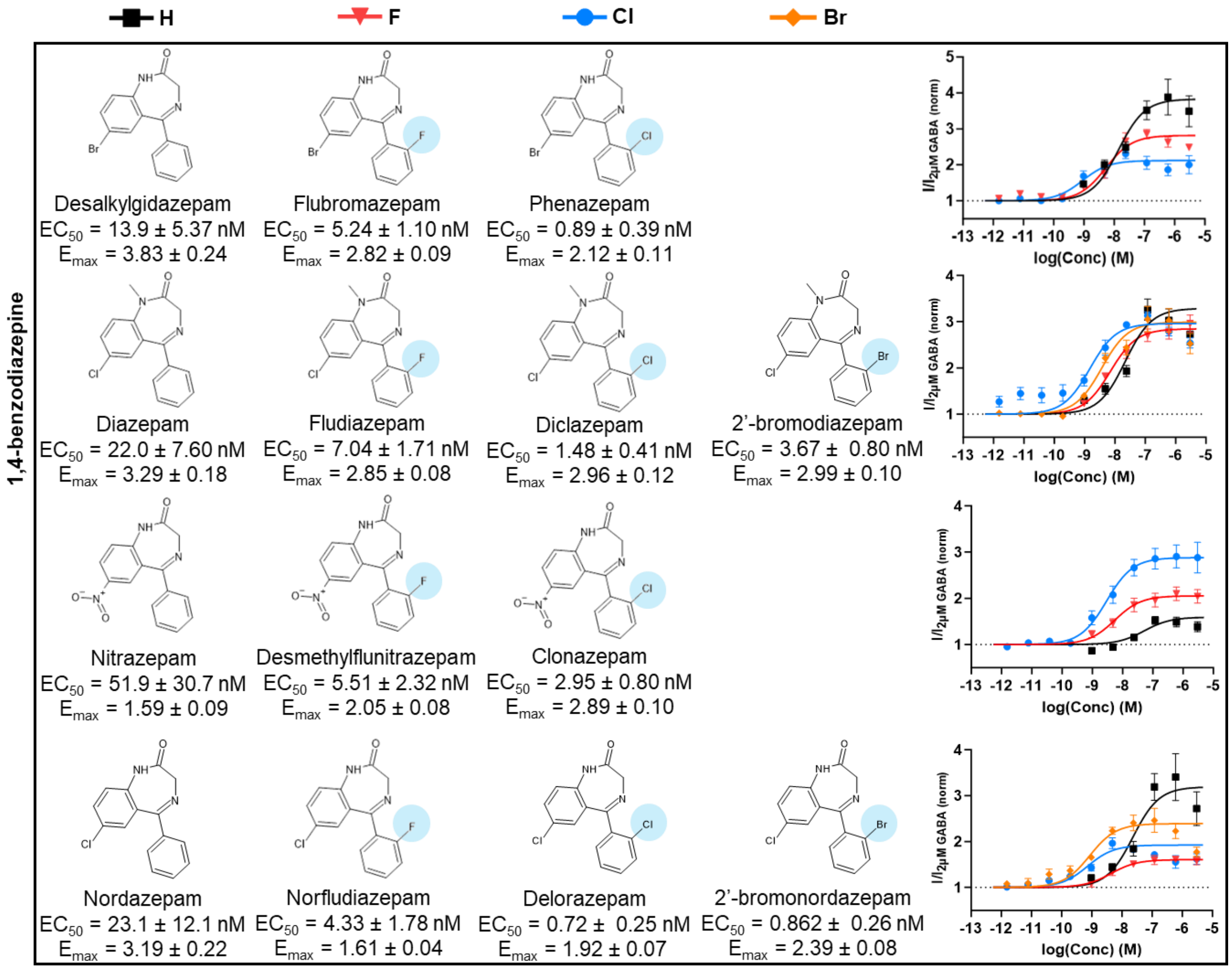

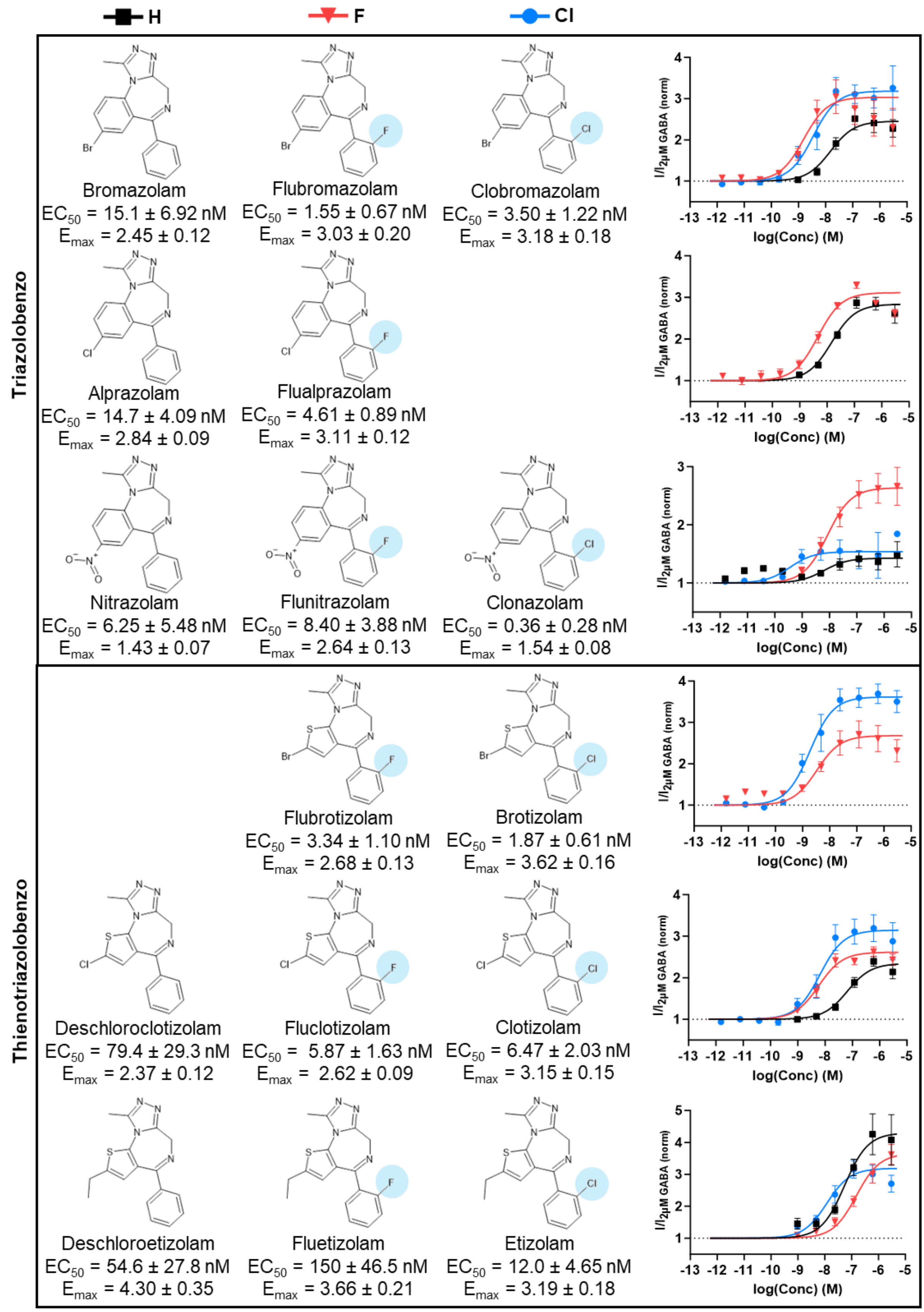

H
F
Cl
Triazolobenzo
Bromazolam
EC_{50} = 15.1 ± 6.92 nM
E_{max} = 2.45 ± 0.12
Flubromazolam
EC_{50} = 1.55 ± 0.67 nM
E_{max} = 3.03 ± 0.20
Clobromazolam
EC_{50} = 3.50 ± 1.22 nM
E_{max} = 3.18 ± 0.18
Alprazolam
EC_{50} = 14.7 ± 4.09 nM
E_{max} = 2.84 ± 0.09
Flualprazolam
EC_{50} = 4.61 ± 0.89 nM
E_{max} = 3.11 ± 0.12
Nitrazolam
EC_{50} = 6.25 ± 5.48 nM
E_{max} = 1.43 ± 0.07
Flunitrazolam
EC_{50} = 8.40 ± 3.88 nM
E_{max} = 2.64 ± 0.13
Clonazolam
EC_{50} = 0.36 ± 0.28 nM
E_{max} = 1.54 ± 0.08
Thienotriazolobenzo
Flubrotizolam
EC_{50} = 3.34 ± 1.10 nM
E_{max} = 2.68 ± 0.13
Brotizolam
EC_{50} = 1.87 ± 0.61 nM
E_{max} = 3.62 ± 0.16
Deschloroclotizolam
EC_{50} = 79.4 ± 29.3 nM
E_{max} = 2.37 ± 0.12
Fluclotizolam
EC_{50} = 5.87 ± 1.63 nM
E_{max} = 2.62 ± 0.09
Clotizolam
EC_{50} = 6.47 ± 2.03 nM
E_{max} = 3.15 ± 0.15
Deschloroetizolam
EC_{50} = 54.6 ± 27.8 nM
E_{max} = 4.30 ± 0.35
Fluetizolam
EC_{50} = 150 ± 46.5 nM
E_{max} = 3.66 ± 0.21
Etizolam
EC_{50} = 12.0 ± 4.65 nM
E_{max} = 3.19 ± 0.18
$I/I_{2\mu M\ GABA}$ (norm)
log(Conc) (M)

SECTION 6

Comparison of BZD parent compounds and metabolites and prodrugs and their primary metabolite

In this section, the structure of each benzodiazepine parent compound, prodrug, and metabolite studied is provided with the structural differences highlighted. The concentration-response curves for the compared benzodiazepines are provided.

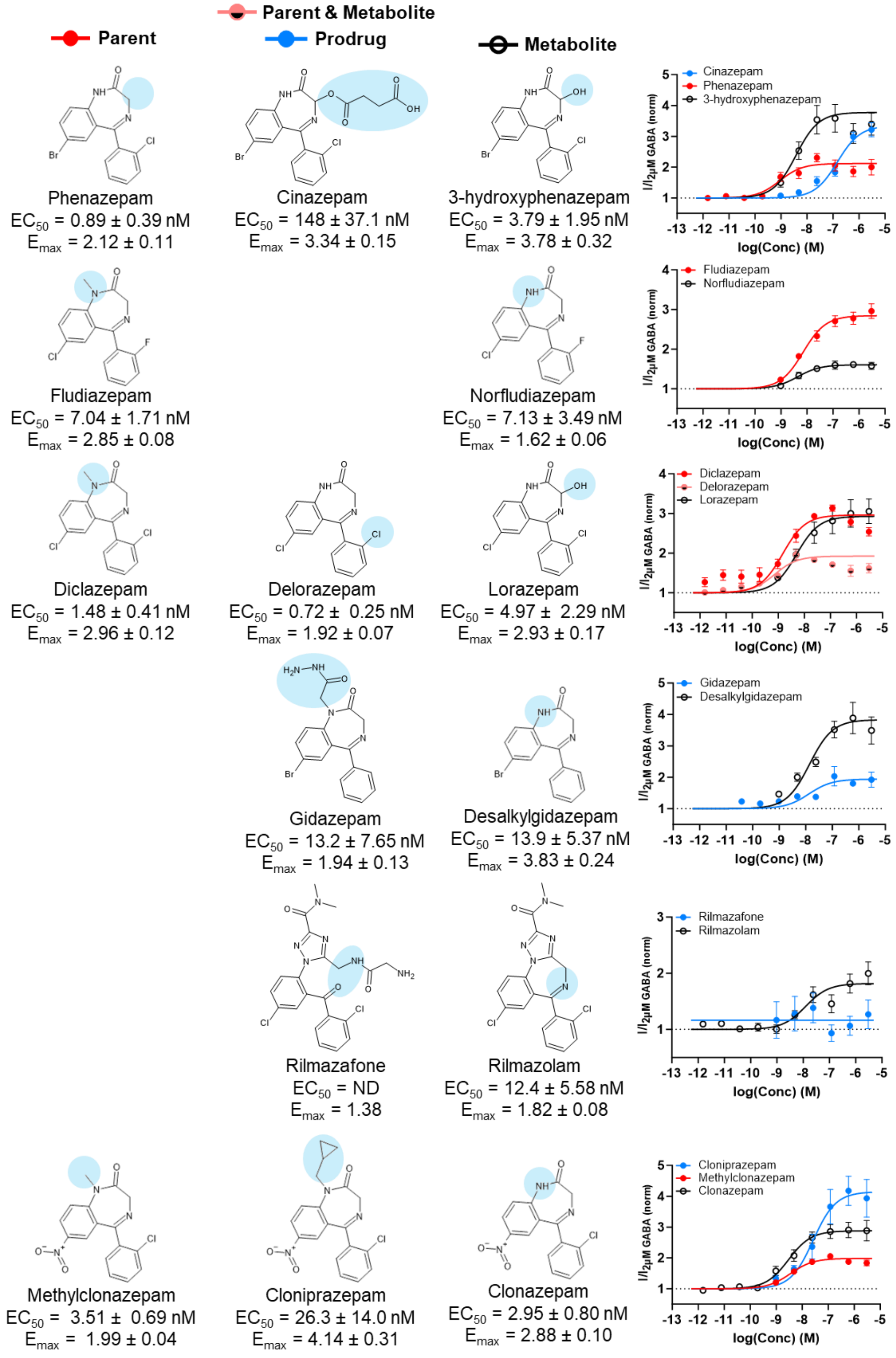

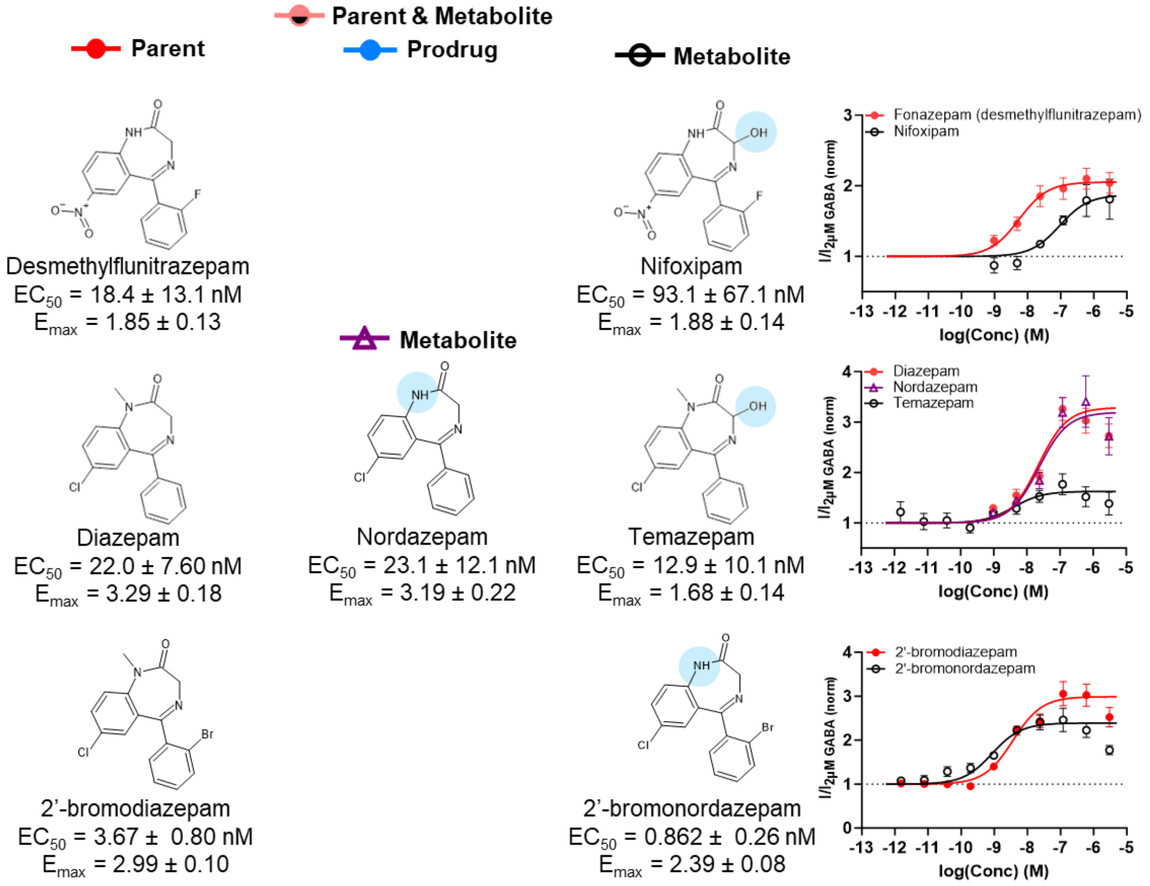

Parent & Metabolite
Parent
Prodrug
Metabolite
Desmethylflunitrazepam
EC50 = 18.4 ± 13.1 nM
Emax = 1.85 ± 0.13
Nifoxipam
EC50 = 93.1 ± 67.1 nM
Emax = 1.88 ± 0.14
Fonazepam (desmethylflunitrazepam)
Nifoxipam
Metabolite
Diazepam
EC50 = 22.0 ± 7.60 nM
Emax = 3.29 ± 0.18
Nordazepam
EC50 = 23.1 ± 12.1 nM
Emax = 3.19 ± 0.22
Temazepam
EC50 = 12.9 ± 10.1 nM
Emax = 1.68 ± 0.14
Diazepam
Nordazepam
Temazepam
2'-bromodiazepam
EC50 = 3.67 ± 0.80 nM
Emax = 2.99 ± 0.10
2'-bromonordazepam
EC50 = 0.862 ± 0.26 nM
Emax = 2.39 ± 0.08
2'-bromodiazepam
2'-bromonordazepam
I/I2μM GABA (norm)
log(Conc) (M)

SECTION 7

Complete data from the statistical analysis of structure-activity relationships

Supplementary Table 1. Statistical comparison from Brown-Forsythe and Welch ANOVA tests ($\alpha = 0.05$) and pairwise t-test for the efficacy (E_{max}) and potency (EC_{50}) between benzodiazepines with different base structures that are close analogs.

	**Comparisons**		**Efficacy (E_{max})**			**Potency (EC_{50})**		
	**Compound 1**	**Compound 2**	**Mean Diff**	**P value**	**Significant?**	**Mean Diff**	**P value**	**Significant?**
**1,4 vs. 1,3,4-triazolo**	Phenazepam	Clobromazolam	-1.066	0.0004	Yes	-0.5949	0.0715	No
	Flubromazepam	Flubromazolam	-0.2163	0.7004	No	0.5286	0.0698	No
	Diazepam	Alprazolam	0.4516	0.1615	No	0.1769	0.7427	No
	Fludiazepam	Flualprazolam	-0.2686	0.2039	No	0.1837	0.4726	No
	Desmethylflunitrazepam	Flunitrazolam	-0.5843	0.0049	Yes	-0.1831	0.8737	No
	Desalkylgidazepam	Bromazolam	1.375	0.0055	Yes	-0.0374	0.9983	No
	Clonazepam	Clonazolam	1.341	<0.0001	Yes	0.9105	0.0632	No
	Nitrazepam	Nitrazolam	0.1643	0.4312	No	0.9200	0.1624	No
	**Pairwise t-test**		**-**	**0.6430**	**No**	**-**	**0.2435**	**No**
**1,4 vs. Thienotriazolo**	Phenazepam	Brotizolam	-1.498	<0.0001	Yes	-0.3225	0.4594	No
	Flubromazepam	Flubrotizolam	0.1377	0.7581	No	0.1960	0.5882	No
	Diclazepam	Clotizolam	-0.1845	0.7152	No	-0.6405	0.0060	Yes
	Diazepam	Deschloroclotizolam	0.9425	0.0065	Yes	-0.4706	0.1008	No
	Fludiazepam	Fluclotizolam	0.2299	0.2413	No	-0.0793	-0.9423	No
	**Pairwise t-test**		**-**	**0.8613**	**No**	**-**	**0.2212**	**No**
**1,3,4-triazolo vs. Thienotriazolo**	Clobromazolam	Brotizolam	-0.4322	0.2234	No	0.2724	0.4885	No
	Flubromazolam	Flubrotizolam	0.3540	0.3842	No	-0.3326	0.4289	No
	Alprazolam	Deschloroclotizolam	0.4909	0.0139	Yes	-0.6475	0.0151	Yes
	Flualprazolam	Fluclotizolam	0.4985	0.0201	Yes	-0.1044	0.8557	No
	**Pairwise t-test**		**-**	**0.3812**	**No**	**-**	**0.3715**	**No**
**ANOVA for all three base structures**			**-**	**0.1669**	**No**	**-**	**0.1858**	**No**

Supplementary Table 2. Statistical comparison from Brown-Forsythe and Welch ANOVA tests ($\alpha = 0.05$) for the efficacy (E_{max}) and potency (EC_{50}) between substitutions at the 4 position on the fused benzene ring (R_4 in Figure 1) of 1,4-benzodiazepines (base A in Figure 1) and 1,3,4-triazolobenzodiazepines (base B in Figure 1) and substitutions on the fused thieno ring (R_5 in Figure 1) of thienotriazolobenzodiazepines (base D in Figure 1).

	**Comparisons**		**Base**	**Efficacy (E_{max})**			**Potency (EC_{50})**		
	**Compound 1**	**Compound 2**		**Mean Diff**	**P value**	**Significant?**	**Mean Diff**	**P value**	**Significant?**
**Cl vs. Br**	Delorazepam	Phenazepam	A	-0.1940	0.3926	No	-0.0900	0.9755	No
	Flubromazepam	Norfludiazepam	A	1.211	<0.0001	Yes	0.0833	0.9635	No
	Alprazolam	Bromazolam	B	0.3828	0.1154	No	-0.01434	0.9999	No
	Flualprazolam	Flubromazolam	B	0.08003	0.9796	No	0.4732	0.1096	No
	Brotizolam	Clotizolam	D	0.4701	0.1168	No	-0.5388	0.0372	Yes
**Cl vs. NO_2**	Clonazepam	Delorazepam	A	0.9562	<0.0001	Yes	-0.6104	0.0134	Yes
	Desmethylflunitrazepam	Norfludiazepam	A	0.4473	0.0011	Yes	0.1051	0.9658	No
	Flualprazolam	Flunitrazolam	B	0.4760	0.0407	Yes	-0.2603	0.5659	No
**Br vs. NO_2**	Phenazepam	Clonazepam	A	-0.7622	0.0002	Yes	-0.5204	0.0919	No
	Desalkylgidazepam	Nitrazepam	A	2.237	0.0008	Yes	-0.5729	0.2492	No
	Flubromazepam	Desmethylflunitrazepam	A	0.7639	<0.0001	Yes	-0.02178	0.9993	No
	Flubromazolam	Flunitrazolam	B	0.3960	0.2985	No	-0.7335	0.0461	Yes
	Clobromazolam	Clonazolam	B	1.645	<0.0001	Yes	0.9850	0.0489	Yes
**Cl vs. CH_2CH_3**	Clotizolam	Etizolam	D	-0.04275	0.9968	No	-0.2671	0.5479	No
**Br vs. CH_2CH_3**	Flubrotizolam	Fluetizolam	D	-0.9774	0.0084	Yes	-1.652	<0.0001	Yes
	Brotizolam	Etizolam	D	0.4273	0.2663	No	-0.8059	0.0129	Yes

Supplementary Table 3. Statistical comparison from Brown-Forsythe and Welch ANOVA tests ($\alpha = 0.05$) for the efficacy (E_{max}) and potency (EC_{50}) between halogen substitutions at the 2 position on the benzene ring (R_6 in Figure 1) of 1,4-benzodiazepines (base A in Figure 1), 1,3,4-triazolobenzodiazepines (base B in Figure 1), and thienotriazolobenzodiazepines (base D in Figure 1).

	**Comparisons**		**Base**	**Efficacy (E_{max})**			**Potency (EC_{50})**		
	**Compound 1**	**Compound 2**		**Mean Diff**	**P value**	**Significant?**	**Mean Diff**	**P value**	**Significant?**
**H vs. F**	Diazepam	Fludiazepam	A	0.4431	0.1535	No	0.4951	0.0668	No
	Nordazepam	Norfludiazepam	A	1.587	0.0207	Yes	0.7268	0.1969	No
	Desalkylgidazepam	Flubromazepam	A	1.011	0.0269	Yes	0.4234	0.1729	No
	Desmethylflunitrazepam	Nitrazepam	A	0.4616	0.0094	Yes	-0.9745	0.0324	Yes
	Alprazolam	Flualprazolam	B	-0.2771	0.2378	No	0.5019	0.0387	Yes
	Bromazolam	Flubromazolam	B	-0.5798	0.0805	No	0.9894	0.0189	Yes
	Flunitrazolam	Nitrazolam	B	1.210	<0.0001	Yes	0.1286	0.9867	No
	Deschloroclotizolam	Fluclotizolam	D	-0.2695	0.1703	No	1.045	0.0007	Yes
	Deschloroetizolam	Fluetizolam	D	0.6430	0.3655	No	-0.4380	0.3170	No
**H vs. Cl**	Diazepam	Diclazepam	A	0.3265	0.4059	No	1.173	0.0002	Yes
	Nordazepam	Delorazepam	A	1.269	0.0222	Yes	1.503	0.0076	Yes
	Desalkylgidazepam	Phenazepam	A	1.711	0.0016	Yes	1.193	0.0012	Yes
	Clonazepam	Nitrazepam	A	1.289	<0.0001	Yes	-1.246	0.0087	Yes
	Bromazolam	Clobromazolam	B	-0.7303	0.0149	Yes	0.6359	0.1014	No
	Clonazolam	Nitrazolam	B	0.1116	0.6292	No	-1.236	0.0638	No
	Deschloroclotizolam	Clotizolam	D	-0.8005	0.0006	Yes	-1.003	-0.0001	Yes
	Deschloroetizolam	Etizolam	D	1.111	0.0697	No	0.6593	0.1132	No
**H vs. Br**	Diazepam	2’-bromodiazepam	A	0.2986	0.6411	No	0.7784	0.0095	Yes
	Nordazepam	2’-bromonordazepam	A	0.8026	0.1016	No	1.427	0.0076	Yes
**F vs. Cl**	Diclazepam	Fludiazepam	A	0.1166	0.8028	No	-0.6774	0.0034	Yes
	Delorazepam	Norfludiazepam	A	0.3180	0.0060	Yes	-0.7766	0.0297	Yes
	Flubromazepam	Phenazepam	A	0.6992	0.0002	Yes	0.7699	0.0077	Yes
	Clonazepam	Desmethylflunitrazepam	A	0.8269	<0.0001	Yes	-0.2713	0.5316	No
	Clobromazolam	Flubromazolam	B	0.1504	0.9215	No	0.3536	0.3965	No
	Clonazolam	Flunitrazolam	B	-1.099	<0.0001	Yes	-1.365	0.0075	Yes

	Comparisons		Base	Efficacy (E_{max})			Potency (EC_{50})		
	**Compound 1**	**Compound 2**		**Mean Diff**	**P value**	**Significant?**	**Mean Diff**	**P value**	**Significant?**
**F vs. Cl**	Fluclotizolam	Clotizolam	D	-0.5311	0.0239	Yes	-0.0424	0.9934	No
	Flubrotizolam	Brotizolam	D	-0.9367	0.0006	Yes	0.2514	0.5286	No
	Fluetizolam	Etizolam	D	0.4681	0.3054	No	1.097	0.0027	Yes
**F vs. Br**	Fludiazepam	2’-bromodiazepam	A	-0.1445	0.5860	No	0.2833	0.1925	No
	Norfludiazepam	2’-bromonordazepam	A	-0.7844	<0.0001	Yes	0.7007	0.0517	No
**Cl vs. Br**	Diclazepam	2-bromodiazepam	A	-0.02792	0.9971	No	-0.3941	0.0580	No
	Delorazepam	2’-bromonordazepam	A	-0.4664	0.0017	Yes	-0.07596	0.9992	No
**NH vs. CH**	Pyrazolam	Bromazolam	B	-0.8430	0.0076	Yes	-0.05639	0.9959	No

Supplementary Table 4. Statistical comparison from Brown-Forsythe and Welch ANOVA tests (α = 0.05) for the efficacy (E_{max}) and potency (EC_{50}) between halogen substitutions at the 4 position on the benzene ring (R_7 in Figure 1) of 1,4-benzodiazepines (base A in Figure 1).

	Comparisons		Efficacy (E_{max})			Potency (EC_{50})		
	**Compound 1**	**Compound 2**	**Mean Diff**	**P value**	**Significant?**	**Mean Diff**	**P value**	**Significant?**
**F vs. Cl**	4’-fluorodiazepam	4’-chlorodiazepam	-0.1739	0.1157	No	0.8003	0.0017	Yes

Supplementary Table 5. Statistical comparison from Brown-Forsythe and Welch ANOVA tests (α = 0.05) for the efficacy (E_{max}) and potency (EC_{50}) between halogen substitutions at the 6 position on the benzene ring (R_8 in Figure 1) of 1,4-benzodiazepines (base A in Figure 1).

	Comparisons		Efficacy (E_{max})			Potency (EC_{50})		
	**Compound 1**	**Compound 2**	**Mean Diff**	**P value**	**Significant?**	**Mean Diff**	**P value**	**Significant?**
**H vs. F**	Fludiazepam	Difludiazepam	0.7563	0.0004	Yes	0.6731	0.0519	No

Supplementary Table 6. Statistical comparison from Brown-Forsythe and Welch ANOVA tests ($\alpha = 0.05$) for the efficacy (E_{max}) and potency (EC_{50}) between benzodiazepines and a known metabolite.

Comparisons		Efficacy (E_{max})			Potency (EC_{50})		
**Parent/Prodrug**	**Metabolite**	**Mean Diff**	**P value**	**Significant?**	**Mean Diff**	**P value**	**Significant?**
Cinazepam	3-hydroxyphenazepam	-0.4309	0.5694	No	1.591	0.0019	Yes
Phenazepam		-1.657	0.0117	Yes	-0.6290	0.1537	No
Fludiazepam	Norfludiazepam	1.239	<0.0001	Yes	0.2116	0.6877	No
Diclazepam	Delorazepam	1.038	<0.0001	Yes	0.3108	0.3197	No
	Lorazepam	0.03272	0.9978	No	-0.5263	0.1816	No
Delorazepam	Lorazepam	-1.005	0.0139	Yes	-0.8371	0.0345	Yes
Desmethylflunitrazepam (Fonazepam)	Nifoxipam	1.803	0.6287	No	-1.228	0.0507	No
Diazepam	Nordazepam	0.09516	0.9804	No	-0.02010	0.9998	No
	Temazepam	1.662	0.0001	Yes	0.7462	0.1422	No
2’-bromodiazepam	2’-bromonordazepam	0.5991	0.0006	Yes	0.6289	0.0038	Yes
Gidazepam	Desalkylgidazepam	-1.201	0.0120	Yes	0.3343	0.4461	No
Cloniprazepam	Clonazepam	1.263	0.0443	Yes	0.9498	0.0381	Yes
Methylclonazepam	Clonazepam	-0.8949	<0.0001	Yes	0.0757	0.9357	No

Supplementary Table 7. Statistical comparison from Brown-Forsythe and Welch ANOVA tests ($\alpha = 0.05$) for the efficacy (I_{min}) and potency (IC_{50}) between DBZDs with a halogen at the 4 position on the benzene ring alone and in the presence of flumazenil.

Comparisons		Efficacy (I_{min})			Potency (IC_{50})		
**Alone**	**With flumazenil**	**Mean Diff**	**P value**	**Significant?**	**Mean Diff**	**P value**	**Significant?**
4’-fluorodiazepam	4’-fluorodiazepam + 3000 nM flumazenil	0.3332	0.1049	No	-0.5047	0.0963	No
4’-chlorodiazepam	4’-chlorodiazepam + 3000 nM flumazenil	-0.03434	0.9650	No	-0.1607	0.7232	No

SECTION 8

Suggested doses for D/BZDs from TripSit

Supplementary Table 8. Suggested dosing of D/BZDs relative to 10 mg of diazepam from TripSit* and the ratio of the dose of the D/BZD to diazepam. The potencies calculated in this study and the ratios to diazepam are also shown for ease of comparison.

Compound	Suggested dosing from TripSit*		Potency	
	Dose	Ratio	EC_{50} (nM)	Ratio
Diazepam	10	1	22.021	1
Alprazolam	0.5	20	14.653	1.50
Bromazolam	1.3-2	5-7.69	15.145	1.45
Brotizolam	0.2	50	1.871	11.8
Clonazepam	0.5	20	2.951	7.46
Diclazepam	1	10	1.48	14.9
Etizolam	1	10	11.966	1.84
Flubromazepam	6	1.67	5.242	4.20
Flubromazolam	0.2	50	1.552	14.2
Lorazepam	1	10	4.973	4.43
Nitrazepam	10	1	51.927	0.424
Nordazepam	10	1	23.1	0.953
Phenazepam	1	10	0.89	24.7
Pyrazolam	1	10	13.301	1.66
Temazepam	20	0.5	3.95	1.63

* Tripsit. Benzodiazepine Dosage Converter. Accessed April 3, 2025. https://benzo.tripsit.me/
