# Supplementary material for: In vitro γ-aminobutyric acid A (GABAA) receptor activity and binding interactions at the α+/γ2– interface of 53 prescription and designer benzodiazepines
Source: Commun Chem. 2026 Apr 7;9:155. doi: 10.1038/s42004-026-02001-x (PMC13068902; doi:10.1038/s42004-026-02001-x)
Supplement: Supplementary file 3 — Description of Additional Supplementary Files [file 42004_2026_2001_MOESM3_ESM.pdf]

Description of Additional Supplementary Files:

File: Supplementary Data 1

Description: contains the activity data used to generate the concentration-response curves and calculate the EC50 and Emax values.

File: Supplementary Data 2

Description: Contains the activity data from experiments in the presence of flumazenil. Some of the data was screening of two concentrations and some are for complete concentration-response curves in the presence of flumazenil.
